# Dopamine D1-like receptor blockade and stimulation decreases operant responding for nicotine and food in male and female rats

**DOI:** 10.1038/s41598-022-18081-3

**Published:** 2022-08-19

**Authors:** Ranjithkumar Chellian, Azin Behnood-Rod, Ryann Wilson, Karen Lin, Grace Wing-Yan King, Marcella Ruppert-Gomez, Alexandria Nicole Teter, Marcelo Febo, Adriaan W. Bruijnzeel

**Affiliations:** Department of Psychiatry, University of Florida, 1149 Newell Dr., Gainesville, FL 32611 USA

**Keywords:** Motivation, Reward

## Abstract

Dopamine has been implicated in the reinforcing effects of smoking. However, there remains a need for a better understanding of the effects of dopamine D1-like receptor agonists on nicotine intake and the role of sex differences in the effects of dopaminergic drugs on behavior. This work studied the effects of D1-like receptor stimulation and blockade on operant responding for nicotine and food and locomotor activity in male and female rats. The effects of the D1-like receptor antagonist SCH 23390 (0.003, 0.01, 0.03 mg/kg) and the D1-like receptor agonist A77636 (0.1, 0.3, 1 mg/kg) on responding for nicotine and food, and locomotor activity were investigated. The effects of SCH 23390 were investigated 15 min and 24 h after treatment, and the effects of the long-acting drug A77636 were investigated 15 min, 24 h, and 48 h after treatment. Operant responding for nicotine and food and locomotor activity were decreased immediately after treatment with SCH 23390. Treatment with SCH 23390 did not have any long-term effects. Operant responding for nicotine was still decreased 48 h after treatment with A77636, and food responding was decreased up to 24 h after treatment. Treatment with A77636 only decreased locomotor activity at the 48 h time point. There were no sex differences in the effects of SCH 23390 or A77636. In conclusion, the D1-like receptor antagonist SCH 23390 reduces nicotine intake and causes sedation in rats. Stimulation of D1-like receptors with A77636 decreases nicotine intake at time points that the drug does not cause sedation.

## Introduction

The use of tobacco products has severe adverse effects on human health. People who use tobacco products are at high risk for various types of cancer and heart disease^1,2^. Tobacco products and nicotine, which is the main psychoactive compound, induce mild euphoria and improve cognition^3,4^. Nicotine addiction is characterized by the compulsive use of tobacco products and negative affective withdrawal signs upon cessation of nicotine use^5,6^. Despite a gradual decline in tobacco use, smoking remains a significant worldwide health burden^7^. In the US, there are about 40 million people who use tobacco products, and worldwide there are 1.3 billion tobacco users^8,9^. Although the use of tobacco cigarettes is on the decline, there has been a substantial increase in the use of electronic cigarettes (e-cigarettes). E-cigarette use among high schoolers doubled from 2017 to 2019^10^. In the US, about 36 percent of 10th graders (12–13 years of age) and 40% of 12th graders (17–18 years of age) have used e-cigarettes. Furthermore, the COVID-19 pandemic has led to an increase in the use of e-cigarettes^11^.

Dopamine plays a critical role in reward learning, motivated behavior, and positive reinforcing effects of drugs of abuse^12,13^. Studies with humans and animals show that dopamine plays a role in the acute rewarding properties of nicotine^5,14^. Dopamine mediates its effect in the brain via D1-like subfamily (D1 and D5 receptors) and D2-like subfamily (D2, D3, and D4 receptors) receptors^15^. Genetic studies point to a role of D1-like and D2-like receptors in smoking^16,17^. Studies with rats indicate that D1-like and D2-like receptor activation contributes to the rewarding effects of nicotine^18–21^. Furthermore, exposure to nicotine induces adaptations in D1-like receptor function, which contribute to the anhedonia associated with nicotine withdrawal^22^. Dopamine D1-like receptor levels are decreased in smokers, and their levels increase upon smoking cessation^23,24^. Systemic administration of nicotine induces dopamine release in the striatum^25^, and striatal dopamine release has widespread effects throughout the brain and plays a critical role in reward-related behaviors^26^.

Clinical studies indicate that there are sex differences in smoking behavior. Nicotine is more reinforcing in males than females, and smoking cues play a greater role in smoking in females than males^27,28^. A positron emission tomography (PET) study also showed that smoking differently affects the dopamine system of male and female smokers^29^. The dopaminergic response to smoking is greater in the ventral striatum of males and in the dorsal putamen of females. Some, but not all, animal studies suggest that there are sex differences in the reinforcing properties of nicotine. Female rats acquire nicotine intake faster, have a higher level of nicotine intake when the response requirements are low and are more motivated to self-administer nicotine under a progressive ratio schedule^30–32^. In contrast, there is no evidence for sex differences in the effects of noncontingent nicotine administration or nicotine self-administration on reward thresholds in the intracranial self-stimulation procedure^33,34^. Despite that sex differences in the rewarding properties of nicotine have been explored, it is unknown if dopamine agonists and antagonists affect nicotine intake differently in males and females. Furthermore, it is not known if a selective D1-like receptor agonist affects operant responding for nicotine in rats. In the present studies, we investigated the role of dopamine signaling in the reinforcing properties of nicotine in male and female rats. The rats were treated with the D1-like receptor antagonist SCH 23390 or the D1-like receptor agonist A77636. We evaluated the acute and delayed effects of these drugs on nicotine intake. It was also determined if SCH 23390 and A77636 affect operant responding for food and locomotor activity.

## Results

### Baseline nicotine self-administration before SHC 23390 treatment

During the first three nicotine self-administration sessions (0.03 mg/kg/inf) nicotine intake and responding on the active lever decreased (Fig. S1A, Nicotine intake, Time F2,28 = 11.943, P < 0.001; Fig. S1B, Active lever, Time F2,28 = 11.872, P < 0.001). The sex of the rats did not affect nicotine intake or responding on the active lever (Nicotine intake, Sex F1,14 = 1.748, NS; Time × Sex 2,28 = 0.454, NS; Active lever, Sex F1,14 = 2.048, NS; Time × Sex 2,28 = 0.556, NS). Responding on the inactive lever did not change over time and was not affected by the sex of the rats (Fig. S1B, Time F2,28 = 0.354, NS; Sex F1,14 = 0.011, NS; Time × Sex 2,28 = 1.449, NS). During the following six sessions (0.06 mg/kg/inf of nicotine), nicotine intake increased and was not affected by the sex of the rats (Fig. S2A, Time F5,70 = 4.256, P < 0.01; Sex F1,14 = 0.95, NS; Time × Sex F5,70 = 1.4, NS). Responding on the active lever increased over time and there was no effect of sex (Fig. S2B, Time F5,70 = 4.435, P < 0.01; Sex F1,14 = 1.06, NS; Time × Sex F5,70 = 1.427, NS). Responding on the inactive lever decreased and there was no effect of sex (Fig. S2B, Time F5,70 = 2.604, P < 0.05; Sex F1,14 = 0.156, NS; Time × Sex F5,70 = 1.456, NS).

### Baseline nicotine self-administration before A77636 treatment

During the first three nicotine self-administration sessions (0.03 mg/kg/inf) nicotine intake and responding on the active lever decreased (Fig. S1C, Nicotine intake, Time F2,32 = 10.617, P < 0.001; Fig. S1D, Active lever, Time F2,32 = 8.986, P < 0.001). The sex of the rats did not affect nicotine intake or responding on the active lever (Nicotine intake: Sex F1,16 = 1.81, NS; Time x Sex 2,32 = 1.427, NS; Active lever: Sex F1,16 = 1.982, NS; Time × Sex 2,32 = 1.415, NS). Responding on the inactive lever did not change over time, and the females responded more on the inactive lever than the males (Fig. S1D, Time F2,32 = 2.524, NS; Sex F1,16 = 7.014, P < 0.05; Time × Sex 2,32 = 1.661, NS). During the following six sessions (0.06 mg/kg/inf) nicotine intake increased and this was not affected by the sex of the rats (Fig. S2C, Time F5,80 = 3.748, P < 0.01; Sex F1,16 = 1.871, NS; Time × Sex F5,80 = 0.859, NS). Responding on the active lever also increased over time, and there was no sex effect (Fig. S2D, Time F5,80 = 4.607, P < 0.01; Sex F1,16 = 1.9, NS; Time × Sex F5,80 = 1.041, NS). Responding on the inactive lever increased over time (Time F5,80 = 3.69, P < 0.01), and the females responded more on the inactive lever than the males (Fig. S2D, Sex F1,16 = 6.934, P < 0.05; Time × Sex F5,80 = 1.085, NS).

### Baseline combined nicotine intake before SCH 23390 and A77636 treatment

It was also determined if there was a sex difference in nicotine intake when the baseline data from the first (SCH 23390) and second experiment (A77636) were combined (prior to testing the D1-like receptor agonist and antagonist). There was a no sex difference in nicotine intake during the first three sessions when the rats self-administered 0.03 mg/kg/inf of nicotine (Fig. S3, Sex F1,32 = 2.934, NS; Time × Sex = F2,64 = 0.199, NS). There was also no sex difference in nicotine intake during the following six days when the rats had access to 0.06 mg/kg/inf of nicotine (Fig. S3, Sex F1,32 = 2.854, NS; Time × Sex = F5,160 = 0.294, NS). Nicotine intake decreased during the first three sessions (0.03 mg/kg/inf; Time F2,64 = 17.221, P < 0.001) and increased during the following six sessions (0.06 mg/kg/inf; Time F5,160 = 7.513, P < 0.001). When days 1–9 were analyzed, there was a trend towards a higher level of nicotine intake in the females (Time F8,256 = 8.183, P < 0.001; Sex F1,32 = 3.567, P = 0.068; Time × Sex = F8,256 = 0.238, NS).

### Experiment 1A: effect of the D1-like receptor antagonist SCH 23390 on operant responding for nicotine

#### 15-min post SCH 23390 treatment

**Figure 1:**
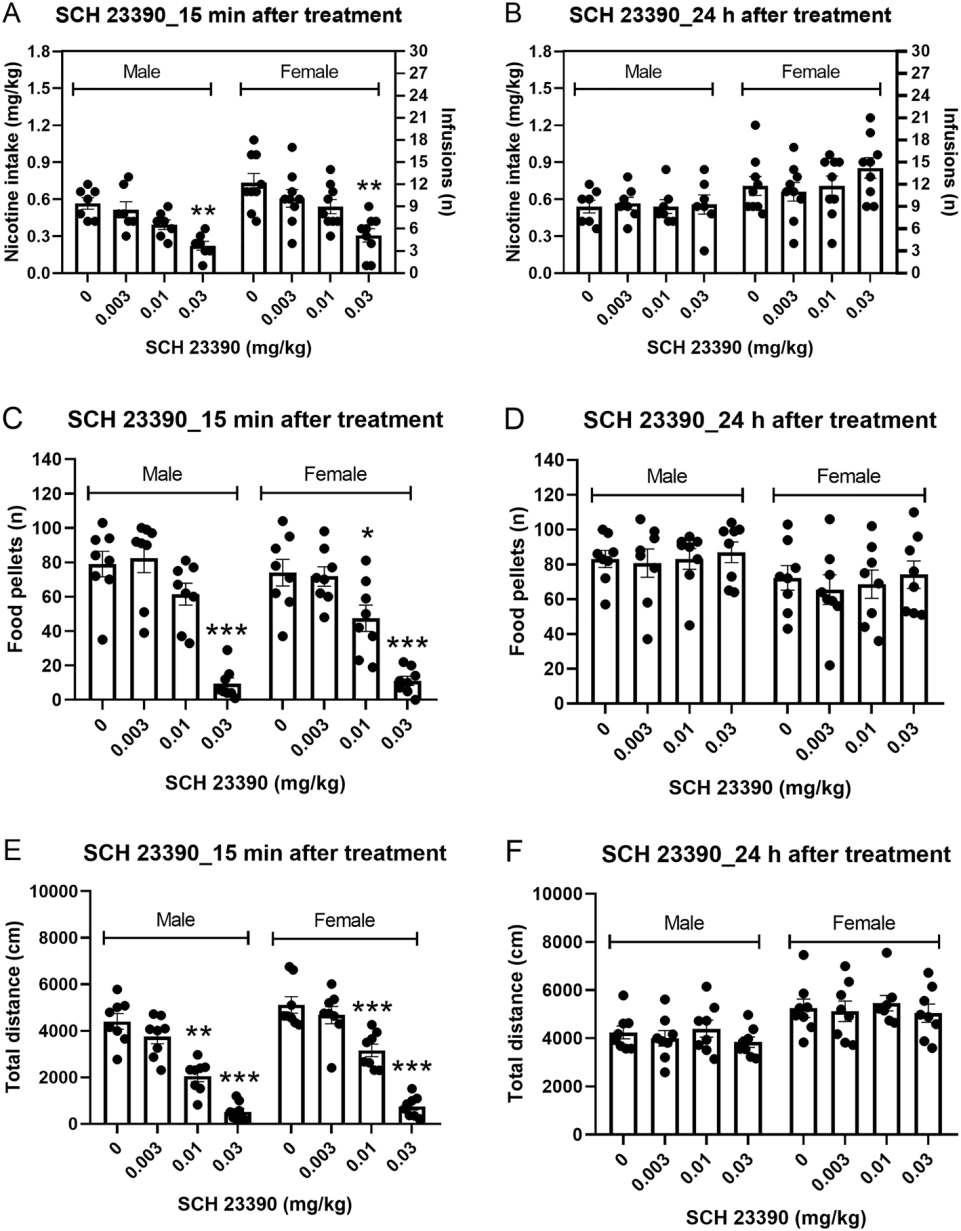
Effects of the D1-like receptor antagonist SCH 23390 on nicotine and food intake and locomotor activity in male and female rats. The effects of SCH 23390 on nicotine (**A** 15 min; **B** 24 h) and food intake (**C** 15 min; **D** 24 h) and locomotor activity (**E** 15 min; **F** 24 h) were investigated. Treatment with SCH 23390 decreased nicotine and food intake and locomotor activity 15 min, but not 24 h, later. Nicotine self-administration (males n = 7, females, n = 9), operant responding for food and small open field test (males n = 8, females, n = 8). Asterisks indicate a significant difference from rats of the same sex that received vehicle. *P < 0.05, **P < 0.01, ***P < 0.001. Data are expressed as means ± SEM.

Nicotine intake was decreased 15-min after treatment with the D1-like receptor antagonist SCH 23390 and there was no effect of sex on nicotine intake (Fig. 1A, Treatment F3,42 = 22.767, P < 0.001; Sex F1,14 = 3.947, NS; Treatment × Sex F3,42 = 0.347, NS). The posthoc test showed that the highest dose of SCH 23390 (0.03 mg/kg) decreased nicotine intake in the male and the female rats. Responding on the active lever was decreased after treatment with SCH 23390 and the females had more active lever presses than the males (Fig. S4A, Treatment F3,42 = 19.312, P < 0.001; Sex F1,14 = 5.676, P < 0.05; Treatment × Sex F3,42 = 0.694, NS). Responding on the inactive lever was decreased after treatment with SCH 23390 and there was no effect of sex (Fig. S4C, Treatment F3,42 = 4.352, P < 0.01; Sex F1,14 = 0.554, NS; Treatment × Sex F3,42 = 0.614, NS).

#### 24-h post SCH 23390 treatment

Treatment with SCH 23390 did not affect nicotine intake 24 h later and there was no effect of sex (Fig. 1B, Treatment F3,42 = 2.114, NS; Sex F1,14 = 3.908, NS; Treatment × Sex F3,42 = 2.022, NS). Responding on the active lever was not affected 24-h after treatment and there was no effect of sex (Fig. S4B, Treatment F3,42 = 1.614, NS; Sex F1,14 = 3.685, NS; Treatment × Sex F3,42 = 1.614, NS). Responding on the inactive lever was not affected after treatment and there was no sex effect (Fig. S4D, Treatment F3,42 = 1.183, NS; Sex F1,14 = 1.431, NS; Treatment × Sex F3,42 = 1.323, NS).

### Experiment 1B: effect of the D1-like receptor agonist A77636 on operant responding for nicotine

#### 15-min post A77636 treatment

**Figure 2:**
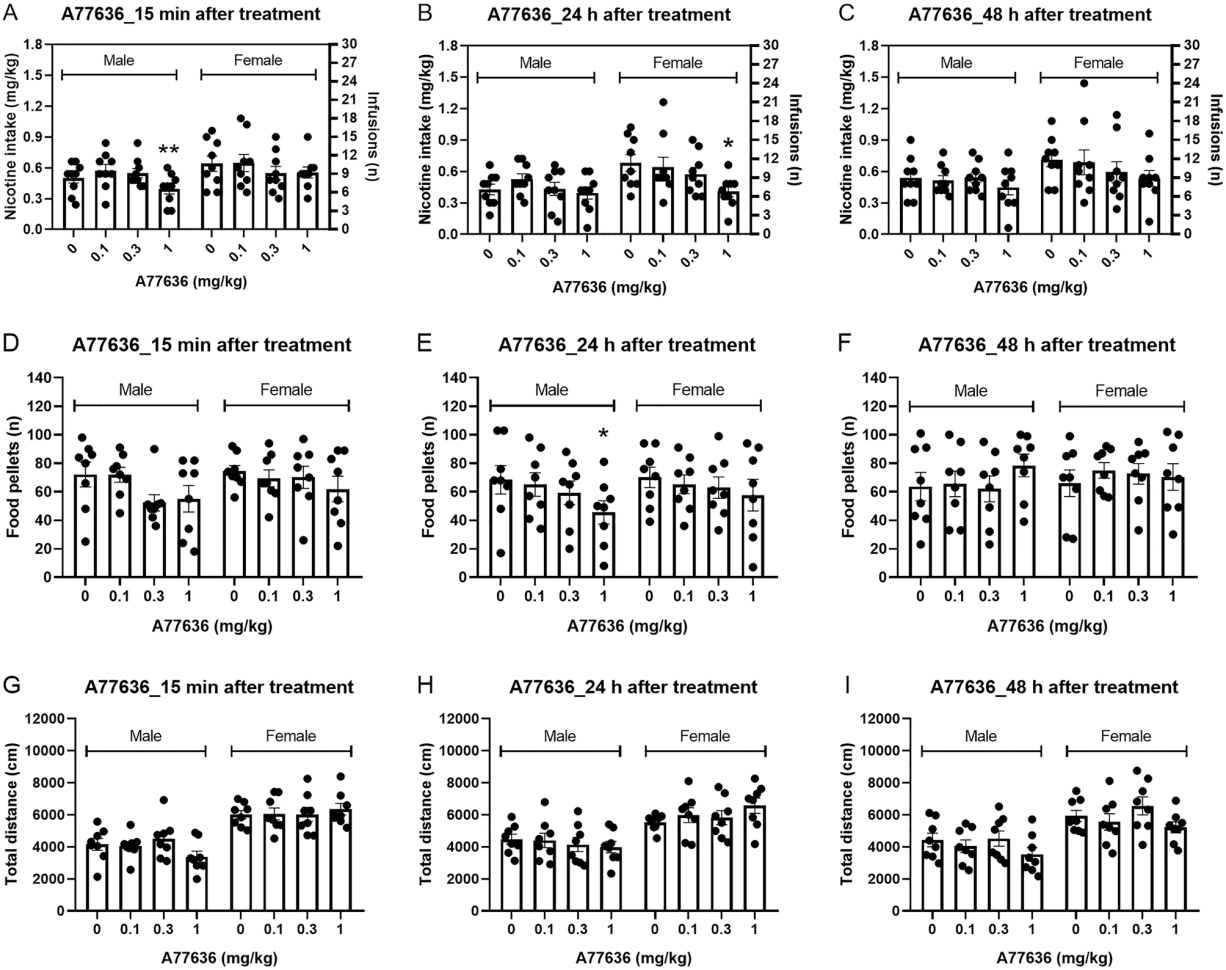
Effects of the D1-like receptor agonist A77636 on nicotine and food intake and locomotor activity in male and female rats. The effects of A77636 on nicotine (**A** 15 min; **B** 24 h; **C** 48 h) and food intake (**D** 15 min; **E** 24 h; **F** 48 h) and locomotor activity (**G** 15 min; **H** 24 h; **I** 48 h) were investigated. Treatment with A77636 decreased nicotine intake 15 min, 24 h, and 48 h later. A77636 decreased food intake at the 15 min and 24 h time point and locomotor activity at the 48 h time point. Nicotine self-administration (males n = 9, females, n = 9), operant responding for food and small open field test (males n = 8, females, n = 8). Asterisks indicate a significant difference from rats of the same sex that received vehicle. *P < 0.05, **P < 0.01. Data are expressed as means ± SEM.

Nicotine intake was decreased 15-min after treatment with A77636 and there was no effect of sex on nicotine intake (Fig. 2A; Treatment F3,48 = 3.631, P < 0.05; Sex F1,16 = 1.799, NS; Treatment × Sex F3,48 = 1.444, NS). The posthoc test showed that 1 mg/kg of A77636 decreased nicotine intake in the males. Responding on the active lever was also decreased after treatment with A77636 and there was no effect of sex (Fig. S5A, Treatment F3,48 = 3.244, P < 0.05; Sex F1,16 = 1.787, NS; Treatment × Sex F3,48 = 1.4, NS). Treatment with A77636 or sex did not affect responding on the inactive lever (Fig. S5D, Treatment F3,48 = 1.472, NS; Sex F1,16 = 0.361, NS; Treatment × Sex F3,48 = 2.711, NS).

#### 24-h post A77636 treatment

Nicotine intake was decreased 24 h after treatment with A77636 and nicotine intake was not affected by the sex of the rats (Fig. 2B, Treatment F3,48 = 5.857, P < 0.05; Sex F1,16 = 3.137, NS; Treatment × Sex F3,48 = 2.204, NS). The posthoc test showed that 1 mg/kg of A77636 decreased nicotine intake in the females. Responding on the active lever was decreased after treatment with A77636 and there was no sex effect (Fig. S5B, Treatment F3,48 = 5.341, P < 0.01; Sex F1,16 = 3.302, NS; Treatment × Sex F3,48 = 2.516, NS). Treatment with A77636 or sex did not affect responding on the inactive lever (Fig. S5E, Treatment F3,48 = 1.557, NS; Sex F1,16 = 0.758, NS; Treatment × Sex F3,48 = 1.018, NS).

#### 48-h post A77636 treatment

Nicotine intake was decreased 48 h after treatment with A77636 and there was no effect of sex on nicotine intake (Fig. 2C, Treatment F3,48 = 3.527, P < 0.05; Sex F1,16 = 1.639, NS; Treatment × Sex F3,48 = 0.942, NS). Responding on the active lever was also decreased after treatment with A77636 and there was no sex effect (Fig. S5C, Treatment F3,48 = 2.883, P < 0.05; Sex F1,16 = 1.855, NS; Treatment × Sex F3,48 = 0.476, NS). Treatment with A77636 or sex did not affect responding on the inactive lever (Fig. S5F, Treatment F3,48 = 0.125, NS; Sex F1,16 = 0.006, NS; Treatment × Sex F3,48 = 2.287, NS).

### Experiment 2A: effect of the D1-like receptor antagonist SCH 23390 on operant responding for food

#### 15-min post SCH 23390 treatment

The number of food pellets received was decreased 15 min after treatment with SCH 23390 and the number of food pellets received was not affected by the sex of the rats (Fig. 1C, Treatment F3,42 = 68.047, p < 0.001; Sex F1,14 = 1.23, NS; Treatment × Sex F3,42 = 0.812, NS). The posthoc test showed that 0.03 mg/kg of SCH 23390 decreased operant responding for food in the male rats and 0.01 and 0.03 mg/kg of SCH 23390 decreased operant responding for food in the female rats. Responding on the active lever was also decreased after treatment with SCH 23390 and not affected by the sex of the rats (Fig. S6A, Treatment F3,42 = 68.002, P < 0.001; Sex F1,14 = 0.821, NS; Treatment × Sex F3,42 = 0.887, NS). Treatment with SCH 23390 or the sex of the rats did not affect responding on the inactive lever (Fig. S6C, Treatment F3,42 = 2.232, NS; Sex F1,14 = 3.515, NS; Treatment × Sex F3,42 = 1.6, NS).

#### 24-h post SCH 23390 treatment

There was no effect of SCH 23390 or sex on the number of food pellets received (Fig. 1D, Treatment F3,42 = 0.849, NS; Treatment × Sex F3,42 = 0.082, NS; Sex F1,14 = 2.611, NS), or active lever responding (Fig. S6B, Treatment F3,42 = 0.866, NS; Sex F1,14 = 2.168, NS; Treatment × Sex F3,42 = 0.083, NS). Treatment with SCH 23390 did not affect inactive lever responding but the females had more inactive lever responses than the males (Fig. S6D, Treatment F3,42 = 1.939, NS; Sex F1,14 = 6.63, P < 0.05; Treatment × Sex F3,42 = 0.083, NS).

### Experiment 2B: effect of the D1-like receptor agonist A77636 on operant responding for food

#### 15-min post A77636 treatment

The number of food pellets received was decreased 15 min after treatment with A77636 and not affected by the sex of the rats (Fig. 2D, Treatment F3,42 = 3.271, p < 0.05; Treatment × Sex F3,42 = 1.199, NS; Sex F1,14 = 0.655, NS). Responding on the active lever was decreased after treatment with A77636 and not affected by the sex of the rats (Fig. S7A, Treatment F3,42 = 3.054, P < 0.05) (Sex F1,14 = 0.633, NS; Treatment × Sex F3,42 = 1.18, NS). Treatment with A77636 or the sex of the rats did not affect responding on the inactive lever (Fig. S7D, Treatment F3,42 = 2.633, NS; Sex F1,14 = 1.151, NS; Treatment × Sex F3,42 = 1.049, NS).

#### 24-h post A77636 treatment

The number of food pellets received was decreased 24 h after treatment with A77636 and there was no effect of sex on the number of food pellets received (Fig. 2E, Treatment F3,42 = 4.701, P < 0.01; Treatment × Sex F3,42 = 0.584, NS; Sex F1,14 = 0.181, NS). The posthoc test showed that 1 mg/kg of A77636 decreased operant responding for food in the males. Responding on the active lever was also decreased after treatment with A77636 and not affected by the sex of the rats (Fig. S7B, Treatment F3,42 = 4.697, P < 0.01; Sex F1,14 = 0.16, NS; Treatment × Sex F3,42 = 0.564, NS). Treatment with A77636 or the sex of the rats did not affect responding on the inactive lever (Fig. S7E, Treatment F3,42 = 1.528, NS; Sex F1,14 = 0.001, NS; Treatment × Sex F3,42 = 0.331, NS).

#### 48-h post A77636 treatment

The number of food pellets received was not affected by treatment with A77636 or the sex of the rats (Fig. 2F, Treatment F3,42 = 1.403, NS; Treatment × Sex F3,42 = 1.525, NS; Sex F1,14 = 0.119, NS). Responding on the active lever was not affected by treatment with A77636 or sex (Fig. S7C, Treatment F3,42 = 1.355, NS; Sex F1,14 = 0.16, NS; Treatment × Sex F3,42 = 1.52, NS). Treatment with A77636 or the sex of the rats did not affect responding on the inactive lever (Fig. S7F, Treatment F3,42 = 0.424, NS; Sex F1,14 = 2.157, NS; Treatment × Sex F3,42 = 1.075, NS).

### Experiment 3A: effect of the D1-like receptor antagonist SCH 23390 on locomotor activity

#### 15-min post SCH 23390 treatment

Treatment with SCH 23390 decreased the total distance traveled and the females traveled a greater distance than the males (Fig. 1E, Treatment F3,42 = 107.826, P < 0.001; Sex F1,14 = 0.767, 8.418, P < 0.05; Sex × Treatment F3, 42 = 1.098, NS). The posthoc test showed that 0.01 and 0.03 mg/kg of SCH 23390 decreased the total distance traveled in the open field in the males and the females. Treatment with SCH 23390 also decreased horizontal beam breaks and the females had the same number of horizontal beam breaks as the males (Fig. S8A, Treatment F3,42 = 117.259, P < 0.001; Sex F1,14 = 0.767, NS; Sex × Treatment F3, 42 = 1.772, NS). SCH 23390 decreased vertical beam breaks and the females had more vertical beam breaks than the males (Fig. S8C, Treatment F3,42 = 70.615, P < 0.001; Sex F1,14 = 18.216, P < 0.001; Sex × Treatment F3,42 = 2.358, NS). SCH 23390 decreased stereotypies and the number of stereotypies was not affected by the sex of the rats (Fig. S8E, Treatment F3,42 = 64.603, P < 0.001; Sex F1,14 = 1.165, NS; Sex × Treatment F3, 42 = 3.929, P < 0.05).

#### 24-h post SCH 23390 treatment

Treatment with SCH 23390 did not affect the total distance traveled 24 h later, and the females traveled a greater distance than the males (Fig. 1F, Treatment F3,42 = 1.196, NS; Sex F1,14 = 10.476, P < 0.01; Sex × Treatment F3,42 = 0.049, NS). Treatment with SCH 23390 and the sex of the rats did not affect horizontal beam breaks (Fig. S8B, Treatment F3,42 = 0.527, NS; Sex F1,14 = 0.566, NS; Sex × Treatment F3,42 = 0.168, NS). Treatment with SCH 23390 did not affect vertical beam breaks and the females had more vertical beam breaks than the males (Fig. S8D, Treatment F3,42 = 1.108, NS; Sex F1,14 = 15.032, P < 0.01; Sex × Treatment F3,42 = 0.058, NS). SCH 23390 and the sex of the rats did not affect stereotypies (Fig. S8F, Treatment F3,42 = 0.43, NS; Sex F1,14 = 0.924, NS; Sex × Treatment F3,42 = 0.279, NS).

### Experiment 3B: effect of the D1-like receptor agonist A77636 on locomotor activity

#### 15-min post A77636 treatment

Treatment with A77636 did not affect the total distance traveled and the females traveled a greater distance than the males (Fig. 2G, Treatment F3,42 = 0.583, NS; Sex F1,14 = 35.041, P < 0.001; Sex × Treatment F3, 42 = 2.106, NS). A77636 did not affect the horizontal beam breaks but the females had more horizontal beam breaks than the males (Fig. S9A, Treatment F3,42 = 2.121, NS; Sex F1,14 = 6.118, P < 0.05; Sex × Treatment F3, 42 = 2.387, NS). A77636 decreased vertical beam breaks and the females had more vertical beam breaks than the males (Fig. S9D, Treatment F3,42 = 3.536, P < 0.05; Sex F1,14 = 21.205, P < 0.001; Sex × Treatment F3,42 = 1.451, NS). A77636 decreased the number of stereotypies and stereotypies were not affected by the sex of the rats (Fig. S9G, Treatment F3,42 = 3.983, P < 0.05; Sex F1,14 = 1.376, NS; Sex × Treatment F3, 42 = 2.686, NS).

#### 24-h post A77636 treatment

A77636 did not affect the total distance traveled and the females traveled a greater distance than the males (Fig. 2H, Treatment F3,42 = 0.571, NS; Sex F1,14 = 13.994, P < 0.01; Sex × Treatment F3, 42 = 2.763, NS). A77636 and the sex of the rats did not affect horizontal beam breaks (Fig. S9B, Treatment F3,42 = 0.285, NS; Sex F1,14 = 2.314, NS;). There was a drug treatment x sex interaction but the post hoc test did not reveal significant effects (Sex × Treatment F3,42 = 3.386, P < 0.05). A77636 decreased vertical beam breaks and the females had more vertical beam breaks than the males (Fig. S9E, Treatment F3,42 = 5.752, P < 0.01; Sex F1,14 = 7.33, P < 0.05; Sex × Treatment F3, 42 = 0.186, NS). A77636 did not affect stereotypies and stereotypies were not affected by the sex of the rats (Fig. S9H, Treatment F3,42 = 0.691, NS; Sex F1,14 = 0.155, NS). There was a drug treatment x sex interaction but the post hoc test did not reveal significant effects (Sex × Treatment F3, 42 = 5.382, P < 0.01).

#### 48-h post A77636 treatment

A77636 decreased the total distance traveled and the females traveled a greater distance than the males (Fig. 2I, Treatment F3,42 = 4.645, P < 0.01; Sex F1,14 = 12.376, P < 0.01; Sex × Treatment F3, 42 = 0.299, NS). Treatment with A77636 decreased horizontal beam breaks and the sex of the rats did not affect the horizontal beam breaks (Fig. S9C, Treatment F3,42 = 8.955, P < 0.001; Sex F1,14 = 1.498, NS; Sex × Treatment F3, 42 = 0.211, NS). Treatment with A77636 decreased vertical beam breaks and the females had more vertical beam breaks than the males (Fig. S9F, Treatment F3,42 = 5.51, P < 0.01; Sex F1,14 = 7.506, P < 0.05; Sex × Treatment F3, 42 = 1.052, NS). A77636 did not affect stereotypies and stereotypies were not affected by the sex of the rats (Fig. S9I, Treatment F3,42 = 2.677, NS; Sex F1,14 = 0.002, NS; Sex × Treatment F3, 42 = 0.71, NS).

## Discussion

In the present study, we investigated the effects of the dopamine D1-like receptor antagonist SCH 23390 and the dopamine D1-like receptor agonist A77636 on operant responding for nicotine and food and locomotor activity in adult male and female rats. Nicotine intake was decreased 15 min, but not 24 h, after the administration of the D1-like receptor antagonist SCH 23390. Nicotine intake was also decreased 15 min, 24 h, and 48 h after the administration of the D1-like receptor agonist A77636. Operant responding for food was decreased 15 min after the administration of SCH 23390, but not 24 h later. Food intake was also decreased 15 min and 24 h, but not 48 h, after the administration of A77636. Locomotor activity was decreased 15 min after the administration of SCH 23390 but not 24 h later. Treatment with A77636 only affected locomotor activity at the 48 h time point. The females traveled a greater distance than the males in the small open field test. The sex of the rats did not affect the effects of SCH 23390 or A77636 on nicotine and food intake and locomotor activity. The present findings show that the D1-like receptor antagonist SCH 23390 induces a brief decrease in nicotine and food intake and that the D1-like receptor agonist A77636 induces a prolonged decrease in nicotine and food intake. The D1-like receptor agonist A77636 decreased nicotine and food intake at time points (15 min and 24-h post treatment) that did not affect locomotor activity.

Evidence indicates that A77636 is a selective dopamine D1-like receptor agonist. A77636 has a higher affinity and selectivity for D1 receptors (inhibition constant (Ki) value = 31 nM) than for D2 receptors (Ki value = 2000 nM) in the rat striatum^35^. Treatment with A77636 induces the release of acetylcholine in the frontal cortex of adult rats, and this effect is completely blocked by treatment with the D1 receptor antagonist SCH 23390^36,37^. Furthermore, SCH 23390 prevents A77636-induced rotational behavior in 6-hydroxydopamine (6-OHDA) lesioned rats. In contrast, pretreatment with the selective D2 antagonist haloperidol did not prevent A77636-induced rotations in 6-OHDA lesioned rats^38^. In the present study, we investigated the effects of the D1-like receptor agonist A77636 on nicotine and food intake and locomotor activity. The D1-like receptor agonist A77636 had a long-term effect on nicotine intake. A77636 decreased nicotine intake 15 min, 24 h and 48 h after treatment. We are not aware of other studies that investigated the effects of A77636 or other D1-like receptor agonists on nicotine self-administration in rats. However, studies have investigated the effects of other D1-like receptor agonists on cocaine self-administration in rats. It has been shown that the D1-like receptor agonists SKF 77434 and SKF 82958 decrease cocaine self-administration in male Wistar rats^39^. Furthermore, low doses of A77636, which do not affect locomotor activity, decrease the cocaine-induced locomotor response in male Swiss Webster mice^40^. These findings suggest that D1-like receptor agonists decrease the reinforcing properties of at least some addictive psychostimulants. Several studies have investigated the effects of D1-like receptor agonists on brain reward function. The dopamine D1-like receptor agonists A77636 and SKF82958 facilitate intracranial self-stimulation (ICSS), which suggests that these compounds have rewarding properties^41,42^. In addition, the dopamine D1-like receptor agonists SKF81297, SKF82958, ABT-431 produced conditioned place preference in rats^43^. However, rhesus monkeys do not self-administer the D1-like receptor agonist SKF 38393^44^. Furthermore, the D1-like receptor agonists SKF 82958 and SKF 77434 do not maintain IV self-administration in rats with a history of cocaine intake^39^. These findings indicate that D1-like receptor agonists decrease drug intake and have some rewarding properties. However, because the D1-like receptor agonists do not maintain self-administration they might have low abuse potential.

Previous research indicates that SCH 23390 is a highly potent and selective dopamine D1-like receptor antagonist. SCH 23390 has a higher affinity and selectivity for D1-like receptors (D1 receptors, Ki value = 0.2 nM; D5 receptors, Ki value = 0.5 nM) than for D2-like receptors (D2 receptors, Ki value = 1100 nM; D3 receptors, Ki value = 800 nM; D4 receptors, Ki value = 3000 nM)^15^. In the present study, we investigated the effects of SCH 23390 on the self-administration of a high dose of nicotine (0.06 mg/kg/inf) in male and female rats. Treatment with the D1-like receptor antagonist led to a large decrease in nicotine intake in the male and the female rats. The present finding is in line with a previous study that investigated the effects SCH 23390 on the self-administration of a lower dose of nicotine (0.03 mg/kg/inf) in adult male Long-Evans rats^18^. Furthermore, it has also been shown that SCH 23390 decreases IV nicotine self-administration in adult female Sprague–Dawley rats^45^. Our work shows that SCH 23390 has a similar effect on nicotine intake in male and female rats. In the present study, we also investigated if SCH 23390 affects operant responding for food and activity in the small open field. We found that SCH 23390 decreases operant responding for food and also decreases activity in the small open field in the males and the females. These findings are in line with previous studies that showed that SCH 23390 dose-dependently decreases operant responding for food and locomotor activity in adult male Long-Evans and Wistar rats^18,46^. The highest dose of SCH 23390 decreased nicotine intake by 50 percent but also caused a large decrease in operant responding for food and locomotor activity. The observation that SCH 23390 induces a large decrease in open field activity suggests that blockade of D1-like receptors has sedative effects or impairs motor function. Indeed, it has been reported that blockade of D1-like receptors in humans and monkeys can cause sedation^47,48^. Overall, the present findings indicate that blockade of D1-like receptors decreases nicotine intake but also causes a decrease in food intake and locomotor activity. The sedative effects of SCH 23390 might mitigate its effectiveness as a smoking cessation treatment.

In the present study, the rats were trained to respond for food pellets before the nicotine self-administration sessions. It is unlikely that food training has a long-term effect on operant responding for nicotine. In a prior study, adult male Wistar rats that were trained to respond for food pellets acquired nicotine self-administration faster than those who were not trained to respond for food pellets (spontaneous acquisition). However, after two sessions, the rats that were food trained and the rats that were not food trained had a similar level of nicotine intake^49^. Furthermore, a study with male Sprague Dawley rats found that training rats to respond for a sucrose solution enhances nicotine intake during the acquisition phase but has not after the acquisition phase^50^. These findings suggests that prior food training has no long-term effect on nicotine intake in rats. In the present study, we also determined sex differences in nicotine intake, food intake, and open-field behavior. The rats self-administered a high dose of nicotine (0.06 mg/kg/inf) for six days before treatment with SCH 23390 or A77636. There was no significant sex difference in baseline nicotine intake, and there was no sex difference in nicotine intake during the experiment. In our previous work with adult Wistar rats and a lower dose of nicotine (0.03 mg/kg/inf), we observed a higher level of nicotine intake in female than male rats^32,33^. However, in rats that were trained to respond on the active lever for food pellets before the nicotine self-administration sessions sex differences were only observed after the acquisition phase (10 days of nicotine self-administration)^33^. Food training leads to a high level of nicotine intake during the acquisition phase, which may mask sex differences in nicotine intake. Other studies reported that there are no sex differences in nicotine intake when nicotine intake has been established, and the rats are tested under FR schedules with low response requirements^31,51–53^. Female rats may self-administer more nicotine than males during the acquisition phase in studies without prior food training^31,54,55^. Furthermore, female rats are more motivated (higher break points) to self-administer nicotine under a progressive ratio schedule^31^. These studies suggest that females tend to self-administer more nicotine than males and these sex differences are most likely to be observed during the acquisition phase and when the response requirements are high. A recent meta-analysis that was based on 20 studies concluded that female rats self-administer more nicotine than male rats^56^. This suggests that large numbers of animals are needed to detect sex difference in nicotine intake in rats. In this study we also compared sex differences in activity in the small open field test. Compared to the males, the females traveled a greater distance and displayed more rearing. This is in line with previous studies that have shown that female rats are more active in the small open field and other behavioral tests^32,57^. We did not observe sex difference in operant responding for food pellets. This is in line with previous work in which we showed that there are no sex difference in operant responding for food pellets in adult Wistar rats when the response requirements are low^58^. Sex difference in operant responding for food are observed when the response requirement are gradually increased using a behavioral economics procedure^58^.

Nicotine has both rewarding and aversive properties. An intermediate dose of nicotine (0.5 mg/kg) induces conditioned place preference in mice but at high dose (2 mg/kg) causes conditioned place aversion^59^. Pretreatment with SCH 23390 or A77636 prevents nicotine (1.75 mg/kg)-induced conditioned place aversion in mice^60^. Similarly, in the present study, both SCH 23390 and A77636 decreased nicotine intake. This suggests that stimulation and blockade of D1-like receptors prevent the motivational effects of nicotine. Treatment with SCH 23390 did not have any long-term effects on nicotine and food intake. Interestingly, treatment with A77636 had a long-term effect on nicotine and food intake. This observation is in line with other studies that showed that A77636 has long-term (> 20 h) anti-Parkinson’s and anorexic effects in rats^38,61^. One study with A77636 (1 mg/kg, SC) showed that this drug induces a robust rotational response in 6-hydroxydopamine (6-OHDA) lesioned rats on the first day of treatment^62^. However, on the second treatment day, the rotation response was greatly decreased, and on the fourth treatment day, the rotation response was similar to the vehicle group^62^. A similar observation was done with the D1-like receptor agonist A68930^63^. On the first treatment day, A68930 caused a robust rotation response, but on the second treatment day, the response was decreased by 95 percent, and on the third treatment day, the response was absent^63^. These behavioral studies indicate that treatment with D1-like receptor agonists can dramatically decrease the sensitivity to D1-like receptor activation. A study with SK-N-MC (neuroblastoma) cells showed that pretreatment with A68930 abolished the cAMP response to subsequent treatment with this drug^35^. Furthermore, pre-incubation of rat striatal membranes with A77636 greatly (76 percent) decreased D1-like receptor binding^35^. Also, A77636 pretreatment reduced D1 receptor binding by 80% and produced desensitization of dopamine-stimulated cAMP accumulation in C-6 glioma cells that were transfected with rhesus macaque dopamine D1A receptors^64^. Similarly, a recent study with HEK293 cells showed that A77636 dose-dependently increases cAMP levels and decreases D1-like receptor levels by almost 50 percent^65^. Therefore, both in vivo and in vitro studies indicate that repeated treatment with A77636 dramatically decreases the sensitivity to D1-like receptor activation. Importantly, clinical studies indicate that there is a positive relationship between D1-like receptor levels and the subjective effects of drugs, and preclinical studies also show a positive association between D1 receptor levels and the behavioral responses to stimulants^66–69^. Taken together, these studies suggest that treatment with A77636 leads to prolonged D1-like receptor desensitization and may thereby decrease the reinforcing properties of nicotine and food.

In the present study, we found that the D1-like receptor antagonist SCH 23390 and the D1-like agonist A77636 decreased operant responding for food in the male and the female rats. The D1-like agonist and antagonist had the same effect on food intake in males and females. Prior studies have reported that SCH 23390 and A77636 decrease food intake in male rats. The D1-like antagonist SCH 23390 decreases operant responding for food in male Wistar and Long Evans rats^18,70^. A study with male Sprague Dawley rats found that the administration of SCH 23390 into the ventral tegmental area decreases responding for food pellets under a progressive ratio schedule^71^. Thus these studies with SCH 23390 indicate that D1-like receptors in the mesolimbic dopaminergic system play a critical role in the reinforcing properties of food. We also found that the D1-like receptor agonist A77636 decreases food intake in male and female rats. A study with male hooded rats showed that A77636 decreased food intake by reducing the meal size and duration^61^. The effect of A77636 on food intake is most likely due to the fact that this drug causes prolonged dopamine D1-like receptor desensitization^35,65^.

In conclusion, the present findings indicate that the D1-like receptor antagonist SCH 23390 induces a brief decrease in nicotine and food intake, and the D1-like receptor agonist A77636 induces a prolonged decrease in nicotine and food intake. However, SCH 23390 doses that decreased nicotine and food intake also caused sedative effects, which may hamper the use of this drug as a smoking cessation aid. The D1-like receptor agonist A77636 decreased nicotine and food intake at doses that did not induce sedative effects. The D1-like receptor agonist A77636 could potentially be used as a smoking cessation aid or appetite suppressant.

## Methods

### Animals

Adult male and female Wistar rats (males 200–250 g, females 175–225 g, 8–9 weeks of age) were purchased from Charles River (Raleigh, NC). Rats were housed with a rat of the same sex in a climate-controlled vivarium on a reversed 12 h light–dark cycle (light off at 7 AM). The rats were gently handled for 2–3 min per day for several days before the food training sessions started. During food training period, the rats were separated, singly housed and remained singly housed for the rest of the study. Food and water were available ad libitum in the home cage except for when the rats were allowed to respond for nicotine or food when they were fed 90–95% of their ad lib home cage intake. The experimental protocols were approved by the University of Florida Institutional Animal Care and Use Committee (IACUC). All experiments were performed in accordance with relevant guidelines and regulations of IACUC and in compliance with the ARRIVE guidelines 2.0 (Animal Research: Reporting of In Vivo Experiments).

### Drugs and treatment

(−)-Nicotine hydrogen tartrate (Sigma-Aldrich), SCH 23390 hydrochloride (Tocris bioscience), and A77636 hydrochloride (Tocris bioscience) were dissolved in sterile saline (0.9% sodium chloride). SCH 23390 and A77636 were administered subcutaneously (SC) in a volume of 1 ml/kg body weight. Nicotine was dissolved in sterile saline, and the rats self-administered 0.03 or 0.06 mg/kg/inf of nicotine in a volume of 0.1 ml/inf. Nicotine doses are expressed as base, and SCH 23390 and A77636 doses are expressed as salt. The treatment schedule was the same in experiments 1–3. Both SCH 23390 and A77636 were administered (SC) 15 min before nicotine self-administration, food responding, or the small open field test. It was also determined if SCH 23390 affected nicotine and food intake and locomotor activity 24 h after treatment and if A77636 affected these parameters 24 and 48 h after treatment. The doses of SCH 23390 and A77636 were based on previous studies in rats^18,61^. SCH 23390 (0, 0.003, 0.01, and 0.03 mg/kg), and A77636 (0, 0.1, and 0.3 mg/kg) were administered according to a Latin square design. The highest dose of A77636, 1 mg/kg, was not included in the Latin square design and was administered last. There was at least 48 h between injections with SCH 23390 and 72 h between injections with A77636^61^.

### Food training

Rats were trained to press a lever for food pellets in the operant chambers (Med Associates, St. Albans, VT). Food training was conducted before the catheters were implanted and also before the rats were treated with SCH 23390 or A77636 in the food studies. Responding on the active lever (right lever, RL) resulted in the delivery of a food pellet (45 mg, F0299, Bio-Serv, Frenchtown, NJ). Responding on the inactive lever (left lever, LL) was recorded but did not have scheduled consequences. Food delivery was paired with a cue light, which remained illuminated throughout the time-out (TO) period. The food training sessions were conducted for 10 days. Instrumental training started under an FR1-TO1s reinforcement schedule for 5 days (30 min session per day). After the fifth food training session, the rats were singly housed and remained singly housed for the rest of the study. On day 6, the time-out period was increased to 10 s. The rats were allowed to respond for food pellets under the FR1-TO10s schedule (20 min sessions) for 5 days. Both levers were retracted during the 10 s time-out period. During the food training period, the rats were fed 90–95% of their baseline food intake in the home cage.

### Intravenous catheter implantation

The catheters were implanted as described before^32,33^. The rats were anesthetized with an isoflurane-oxygen vapor mixture (1–3%) and prepared with a catheter in the right jugular vein. The catheters consisted of polyurethane tubing (length 10 cm, inner diameter 0.64 mm, outer diameter 1.0 mm, model 3Fr, Instech Laboratories, Plymouth Meeting, PA). The right jugular vein was isolated, and the catheter was inserted to a depth of 3 cm for males and 2.5 cm for females. The tubing was then tunneled subcutaneously and connected to a vascular access button (Instech Laboratories, Plymouth Meeting, PA). The button was exteriorized through a small, 1-cm incision between the scapulae. After the surgery, the rats were given at least seven days to recover. The rats received daily IV infusions of the antibiotic Gentamycin (4 mg/kg, Sigma-Aldrich, St. Louis, MO) for seven days. A sterile heparin solution (0.1 ml, 50 U/ml) was flushed into the catheter before and after administering the antibiotic or nicotine self-administration. Then 0.05 ml of a sterile heparin/glycerol lock solution (500 U/ml) was infused into the catheter. The animals received carprofen (5 mg/kg, SC) daily for 48 h after the surgery. One day before nicotine self-administration, the rats were allowed to respond for food pellets under the FR1-TO10s schedule (20-min session).

### Experiment 1: effects of SCH 23390 and A77636 on nicotine self-administration

The rats were allowed to self-administer nicotine for nine daily 1 h sessions (baseline). During the first three sessions (days 1–3), the rats self-administered 0.03 mg/kg/inf of nicotine under an FR1-TO10s schedule. For the following six sessions (days 4–9) the rats self-administered 0.06 mg/kg/inf of nicotine under an FR1-TO60s schedule. The 0.06 mg/kg/inf dose of nicotine is a relatively high dose and leads to high levels of nicotine intake^52,72^. During the first day of each dose the amount of nicotine that the rats could self-administer was limited to prevent aversive effects. The maximum number of infusion was set to 20 for the first day that the rats received the 0.03 mg/kg/inf dose and to 10 for the first day that the rats received the 0.06 mg/kg/inf dose. Active lever (right lever, RL) responding resulted in the delivery of a nicotine infusion (0.1 ml infused over a 5.6-s period). The initiation of the delivery of an infusion was paired with a cue light, which remained illuminated throughout the time-out period. Inactive lever (left lever, LL) responses were recorded but did not have scheduled consequences. Both levers were retracted during the time-out period. After the baseline sessions, the effects of SCH 23390 (Expt. 1A; males n = 9, females n = 9) and A77636 (Expt. 1B; males n = 9, females n = 9) on nicotine self-administration were investigated in daily 1 h sessions (0.06 mg/kg/inf; FR1-TO60s schedule). Before the SCH 23390 and A77636 treatments, the rats received a minimum of 5 infusions per session and had stable levels of nicotine intake (less than 25% variation in nicotine intake across three consecutive days). During the self-administration period, the rats received about 90–95% of their normal ad lib food intake in the home cage. The rats were fed immediately after the operant sessions. A mild level of food restriction facilitates food training and nicotine self-administration in rats^49,73^. Catheter patency was assessed after the last self-administration session by infusing 0.2 ml of the ultra-short action barbiturate Brevital (1% methohexital sodium) in the catheter. Rats with patent catheters displayed a sudden loss of muscle tone. If the rats did not respond to Brevital, their self-administration data were excluded from the analysis. Two animals (male-SCH 23390 group) did not respond to the Brevital, and their self-administration data were excluded from the data analysis (seven male-SCH 23390 rats were included in the data analysis).

### Experiment 2 and 3: effects of SCH 23390 and A77636 on food responding and motor activity

After the food training sessions, the effects of SCH 23390 (Expt. 2A; males n = 8, females n = 8) and A77636 (Expt. 2B; males n = 8, females n = 8) on operant responding for food pellets was studied in daily 20-min sessions under an FR1-TO10s schedule (Expt. 2). The rats were fed 90–95% of their baseline food intake in the home cage after operant responding for food. After the food study, the rats were fed ad-lib, and the effects of SCH 23390 and A77636 on activity parameters in the small open field were investigated (Expt. 3). The rats were habituated to the small open field on three consecutive days (20-min sessions) and then the effects of SCH 23390 and A77636 on locomotor activity, rearing, and stereotypies in the small open field were investigated in 20-min sessions.

### Small open field test

The small open field test was conducted as described before^32,74^. The small open field test was conducted to assess locomotor activity, rearing, and stereotypies. These motor behaviors were measured using an automated animal activity cage system (VersaMax Animal Activity Monitoring System, AccuScan Instruments, Columbus, OH, USA). Horizontal beam breaks and total distance traveled reflect locomotor activity, and vertical beam breaks reflect rearing. The distance traveled is dependent on the path of the animal in the open field and is therefore considered a better indicator of locomotor activity than horizontal beam breaks. Repeated interruptions of the same beam are a measure of stereotypies (stereotypy count)^75^. The setup consisted of four animal activity cages made of clear acrylic (40 cm × 40 cm × 30 cm; L × W × H), with 16 equally spaced (2.5 cm) infrared beams across the length and width of the cage. The beams were located 2 cm above the cage floor (horizontal activity beams). An additional set of 16 infrared beams was located 14 cm above the cage floor (vertical activity beams). All beams were connected to a VersaMax analyzer, which sent information to a computer that displayed beam data through Windows-based software (VersaDat software). The small open field test was conducted in a dark room, and the cages were cleaned with a Nolvasan solution (chlorhexidine diacetate) between animals. Each rat was placed in the center of the small open field, and activity was measured for 20 min.

### Statistics

Baseline nicotine intake was analyzed with a two-way ANOVA with sex as a between subjects factor and time as a within subjects factor. The main goal of this work was to investigate the effects of SCH 23390 and A77636 on operant responding for nicotine and food and locomotor activity in male and female rats. The effects of SCH 23390 and A77636 on operant responding for nicotine and food and open-field behavior were analyzed with a two-way ANOVA with sex as a between subjects factor and drug treatment as a within subjects factor. Separate ANOVA analyses were conducted for the 24 h (SCH 23390 and A77636) and 48 h (A77636) time points to determine if there were delayed effects of SCH 23390 and A77636 treatment. For all statistical analyses, significant effects in the ANOVA were followed by Bonferroni's posthoc tests to determine which groups differed from each other. P-values that were less or equal to 0.05 were considered significant. Significant main effects, interaction effects, and posthoc comparisons are reported in the “Results” section. Data were analyzed with SPSS Statistics version 28 and GraphPad Prism version 9.3.1.

## Supplementary Information

Supplementary Figures.

## Data Availability

Data are available from the corresponding author on request.
